# Hypnotism

**Published:** 1890-02-01

**Authors:** 


### HYPNOTISM.

In The Hospital for March 23, 1889, we reviewed Dr. Tuckey's work on the treatment by "sleep and suggestion" and in The Hospital, December 14, 1889, we commented on cases reported by Dr. Tuckey. The accepted theory of which Dr. Tuckey (in this country, at least) may be regarded as the exponent, is that suggestions made during the hypnotic state are capable not only of affecting the actions of the individual during the hypnotism, but also of affecting the functions of organic life so as to alter a diseased process without the patient being conscious that any suggestion has been made.

Dr. H. C. Hood, of the University of Pennsylvania, has some remarks "On Hypnotism in Therapeutics without Suggestion" (*Lancet*, January 11). Dr. Hood reports several cases, which appear to show that it is not necessary the hypnotiser should speak to the patient specifically about the ailment. "They come to be cured, and they have faith, and if curable they recover without suggestion." But Dr. Hood remarks: "My observations led me to the conclusion that the good results achieved were usually in hysterical cases." As we know, men may be hysterical as well as women. Hysteria, no doubt, covers a wide surface. Neither hypnotism nor suggestion will cure an organic disease. Hypnotism is a revival of what our fathers termed animal magnetism. In 1826 a somnambulist was tried in consequence of the death of a patient treated by prescriptions given during the magnetic sleep. It was then remarked, "That the children of Æsculapius should lose some of their practice (by the resort of patients to magnetisers) is a misfortune with which they must console themselves. But that society should be deprived of one of its members by the audacious ignorance of Messieurs or Mesdames the somnambulists, is too serious an affair to be passed by unnoticed or unpunished." Animal magnetism, mesmerism, hypnotism, by whichever name it is called, may possibly be productive of benefit in some cases, when applied by the educated scientific physician. In the hands of the charlatan it is calculated to promote evil.

